# Conversion efficiency of flight power is low, but increases with flight speed in the migratory bat *Pipistrellus nathusii*

**DOI:** 10.1098/rspb.2023.0045

**Published:** 2023-05-10

**Authors:** Shannon E. Currie, L. Christoffer Johansson, Cedric Aumont, Christian C. Voigt, Anders Hedenström

**Affiliations:** ^1^ Department of Evolutionary Ecology, Leibniz Institute for Zoo and Wildlife Research, 10315 Berlin, Germany; ^2^ Department of Biology, Lund University, 223 62 Lund, Sweden; ^3^ Agrocampus-Ouest, 35042 Rennes, France

**Keywords:** metabolic power input, mechanical power output, energy efficiency, particle image velocimetry, ^13^C-labelled sodium bicarbonate, wind tunnel

## Abstract

The efficiency with which flying animals convert metabolic power to mechanical power dictates an individual's flight behaviour and energy requirements. Despite the significance of this parameter, we lack empirical data on conversion efficiency for most species as *in vivo* measurements are notoriously difficult to obtain. Furthermore, conversion efficiency is often assumed to be constant across flight speeds, even though the components driving flight power are speed-dependent. We show, through direct measurements of metabolic and aerodynamic power, that conversion efficiency in the migratory bat (*Pipistrellus nathusii*) increases from 7.0 to 10.4% with flight speed. Our findings suggest that peak conversion efficiency in this species occurs near maximum range speed, where the cost of transport is minimized. A meta-analysis of 16 bird and 8 bat species revealed a positive scaling relationship between estimated conversion efficiency and body mass, with no discernible differences between bats and birds. This has profound consequences for modelling flight behaviour as estimates assuming 23% efficiency underestimate metabolic costs for *P. nathusii* by almost 50% on average (36–62%). Our findings suggest that conversion efficiency may vary around an ecologically relevant optimum speed and provide a crucial baseline for investigating whether this drives variation in conversion efficiency between species.

## 1. Introduction

Powered flapping flight has evolved in only one mammalian order—the Chiroptera. Although our understanding of the aerodynamics of bat flight has improved considerably in the last 15 years [1–3], relatively little empirical data regarding the cost of flight exist for bats (but see [4–8]). As flight is energetically costly, selection is expected to work to optimize flight performance, including not only the aerodynamics but also the efficiency of converting muscle metabolic power into mechanical power output. Conversion efficiency has the potential to impact individual flight behaviours and flight strategies as it determines the metabolic cost of flight together with aerodynamics. With more than 1400 species, bats exhibit great variability in flight behaviour, ranging from hovering to hawking and long-distance migration [9]. Determining conversion efficiency during different flight modes is thus important to understand the potentials and limitations of bat flight.

During flight, metabolic power fuels muscle work that is converted into the mechanical power required to keep animals aloft, i.e. produce a forward and upward aerodynamic force to overcome drag and gravity [10]. Owing to the intrinsic difficulty in measuring power output in airborne animals, models based on aerodynamic theory are often used to calculate mechanical power at varying air speeds and to infer details regarding optimal flight performance [10–12]. Original models are adapted from aircraft aerodynamic theory and underlying assumptions may be violated when these models are transferred to dynamic and complex biological systems. While flight models have been shown to be reasonably robust in their application to birds [13,14], little empirical evidence exists for bats (but see [15]). Importantly, metabolic power input is often estimated from these models using a fixed value for energy conversion efficiency of muscles. Most studies assume this to be constant across flight speeds and body masses at 23%, a value derived from two landmark studies of birds flying at various angles of tilt in a wind tunnel [16,17]. There are, however, arguments as to why the conversion efficiency should not be constant but rather vary with flight speed, among other factors [18]. If conversion efficiency is not a constant, it is reasonable to assume that evolution has optimized the conversion efficiency to speeds of most ecological relevance for the animals.

In two studied bat species, the maximum range speed (*U*_mr_ i.e. the speed of maximum effective lift to drag ratio, [19]) coincides with the speed of minimum average angular velocity of the flapping wings [20]. The flapping motion of the wing, during the downstroke, is caused mainly by the contraction of the pectoralis muscle, which connects to the humerus at a distance from the shoulder joint. Hence, the distance the muscle contracts determines the angular amplitude of the wing motion, which together with wingbeat frequency determines the angular velocity of the wing [20]. Since the contraction velocity of the flight muscles determines the efficiency of the muscles [10], this suggests that conversion efficiency likely varies with speed. In the cockatiel (*Nymphicus hollandicus*), pectoralis muscle conversion efficiency was estimated from measures of metabolic and mechanical power in a single experimental group and was found to increase with increasing air speed [21]. This was further supported by a meta-analysis that confirmed an increase in calculated conversion efficiency with increasing flight speed by up to 7% across eight species of bats [22].

Although the need for data on conversion efficiency collected using both physiological and mechanical techniques in the same individuals has been widely acknowledged for many years [18,23], few studies have accomplished this in either birds or bats. This has primarily been due to the fact that measuring metabolic rate in flying animals has required techniques that may inhibit natural flight behaviour and that increase drag, such as animals wearing a respirometry mask (for example [7]). Similarly, measures of mechanical power via muscle dynamics (for example [21,24]) may also alter flight performance and aerodynamics owing to their invasive nature and the need for animals to carry loggers. As a consequence, our understanding of the true efficiency of power conversion and how this varies with flight speed remains sparse.

Whole-animal conversion efficiency has only recently been measured directly and non-invasively from the same group of individuals in two vertebrate species, the 18 g fruit bat *Carollia perspicillata* [25] and the 19 g bird *Sylvia atricapilla* [26]*.* Muscle conversion efficiency in *S. atricapilla* was calculated to be around 30% at two air speeds (6 and 8 m s^−^^1^), greater than the 23% assumed for bird flight. On the contrary, whole-animal conversion efficiency was found to vary between 5.9 and 9.8% in *C. perspicillata* across five air speeds (3–7 m s^−^^1^). However, the variation in data was too large to draw firm conclusions about the relationship between conversion efficiency and air speed. These results suggest either that birds are more efficient than bats at converting energy to mechanical power output during flight, or that additional selection pressures originating from different life-history trade-offs of the studied species drive conversion efficiency. One important facet of bat flight is echolocation, which is considered to pose no additional cost to flight [27], at least at nominal intensity. Yet, at high intensities, additional metabolic costs of echolocation may explain a reduction in reported overall conversion efficiency for bats compared with birds [28]. Furthermore, migratory species may be particularly efficient fliers owing to selection pressures associated with migration that require economic use of fuel [29]. *Sylvia atricapilla* is known to engage in long-distance migration, whereas *C. perspicillata* is non-migratory, which could also influence previous findings.

Here we test the assumption of a constant conversion efficiency by directly measuring both metabolic power input and mechanical power output in Nathusius' pipistrelle (*Pipistrellus nathusii*). We do this for the same group of individuals flying freely in a wind tunnel across a range of flight speeds. We examine the role of ecology on overall conversion efficiency by focusing on a migratory bat species. We hypothesized that *P. nathusii* would be more efficient fliers than previously studied bats owing to selection pressures associated with migration, as this species holds the record for longest documented migratory distance in a bat [30]. We also conducted a meta-analysis of the literature available for metabolic rate in birds and bats collected across numerous air speeds, to further compare conversion efficiency between these two taxa.

## 2. Results

We trained 10 *P. nathusii* (four male, six female) to fly in a wind tunnel at air speeds between 4 and 9 m s^−1^. We measured metabolic power input (*P*_met_) using the ^13^C-labelled sodium bicarbonate method [31] and mechanical power output (*P*_mech_) using tomographical particle image velocimetry (TomoPIV) [6]. Metabolic power output data from eight individuals at flight speeds (*U*_∞_) between 3.8 and 8.8 m s^−1^ satisfied our selection criteria (see Methods) and were incorporated in further analyses. Flight mechanical power was quantified for four of these individuals at flight speeds between 5.2 and 9.0 m s^−1^. We assumed that power followed a U-shaped curve with flight speed, as predicted by theory and supported by previous findings for this species [5], and fitted a nonlinear mixed effects model following the generic aerodynamic model of Pennycuick [10]:

2.1 $$P=\;k_{1}U_{\infty }^{3}+\;\frac{k_{2}}{U_{\infty }}+\;k_{3},$$

where *U*_∞_ is air speed, and each coefficient (*k_n_*) represents the aerodynamic power components parasite power (*P*_par_), induced power (*P*_ind_) and profile power (*P*_pro_), respectively.

### (a) Mechanical power output

Estimated *P*_mech_ ranged from 0.046 to 0.140 W with an average of 0.092 ± 0.0031 W (mean ± s.e.m.). A U-shaped curve fitted the data better than a linear mixed effects model (likelihood ratio test *p* < 0.001) when profile power (*P*_pro_) was predefined. Following Pennycuick's [19] argument that *P*_pro_ is proportional to the sum of *P*_par_ and *P*_ind_ at minimum power speed, we determined *P*_pro_ as 50% of the mechanical power at minimum power speed, *U*_mp_. We calculated *U*_mp_ to be 4.6 m s^−1^ using the *animal flight performance tool* (*afpt*; [12,32]) and calculated a *P*_pro_ of 0.0297 W from the mean power at 5 m s^−1^. The model fitted to our data was

2.2 $$P_{\mathrm{mech}}=9.21\times 10^{-5}\;U_{\infty }^{3}+\frac{0.176}{U_{\infty }}+0.0297.$$

**Figure 1.**
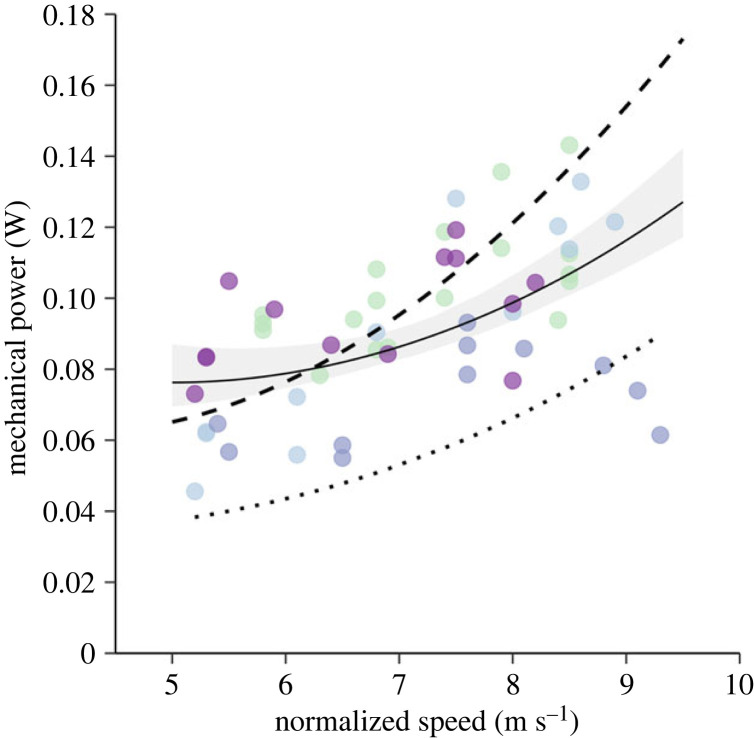
Flight mechanical power measured from the wake. Mechanical power (W) measured from TomoPIV data of four *Pipistrellus nathusii* (individuals represented by different colours) flying in a wind tunnel, alongside aerodynamic model predictions for this species (solid line—mixed effects model fit, dashed line—*afpt*, dotted line—*Flight*). The shaded area illustrates the 95% confidence intervals around our fitted mixed effects model, showing that Pennycuick's *Flight* model [10] underestimates mechanical power in *P. nathusii*, while *afpt* [12] better captures how power varies with flight speed.

Compared with model predictions of *P*_mech_ made from the *Flight* model [10], our data were 0.035 ± 0.003 W higher on average. Compared with the prediction from the *afpt* model, our data differed by only 0.011 ± 0.004 W on average, and the *afpt* more closely predicted *P*_mech_ at higher air speeds (figure 1). See electronic supplementary material, figure S1 for an example of the vortex structure of *P. nathusii*.

After normalizing power output values by lift, to make them comparable to weight-specific measures, our model fitted to the data was

2.3 $$P_{\mathrm{mech}/L}=9.03\times 10^{-4}\;U_{\infty }^{3}+\;\frac{2.22}{U_{\infty }}+0.391.$$

We calculated minimum power speed (*U*_mp,mech_) to be 5.2 m s^−1^ from the model fitted to our mechanical power measurements. This is higher than the minimum power speed predicted from aerodynamic theory using the *Flight* model (3.6 m s^−1^) or using the *afpt* (4.6 m s^−1^). Our model fitted to lift-specific values resulted in a *U*_mp,mech/L_ of 5.5 m s^−1^.

Maximum range speed (*U*_mr_) predicted from aerodynamic theory was 6.9 m s^−1^ using the *Flight* model and 6.0 m s^−1^ from the *afpt*, similar to the *U*_mr,mech_ calculated from the model fitted to our data (6.8 m s^−1^). The lift-specific maximum range speed (*U*_mr,mech/L_) increased only slightly, to 7.3 m s^−1^.

### (b) Metabolic power input

**Figure 2.**
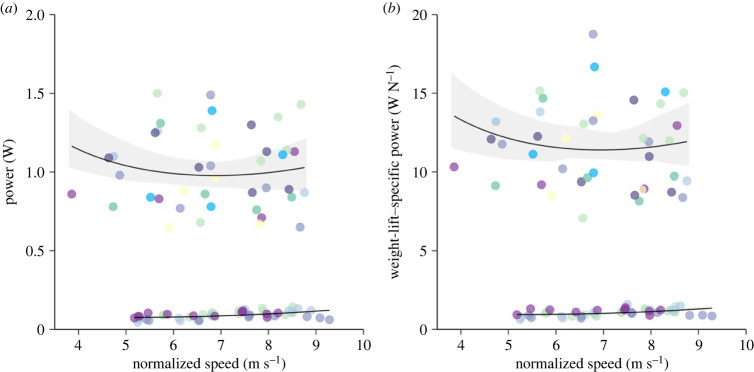
Flight power in Nathusius' pipistrelles, *Pipistrellus nathusii*. Metabolic and mechanical power (W) in relation to air speed (m s^−1^) for (*a*) raw data and (*b*) weight- or lift-specific data from a total of eight *P. nathusii*. Metabolic power was measured in eight individuals (represented by different colours), while mechanical power was only measured in four of these individuals. Solid lines and grey shading indicate our mixed effects model fits with 95% confidence intervals.

The average *P*_met_ in flight across all speeds was 1.0 ± 0.04 W and ranged from an absolute minimum of 0.65 W for a bat flying at 8.7 m s^−1^ up to 1.5 W for an individual flying at 5.7 m s^−1^ (figure 2*a*). As for *P*_mech_, we fitted both linear and nonlinear mixed effects models. The constant in the nonlinear model was set to 50% of the metabolic power at minimum power speed (i.e. 5 m s^−1^ as *U*_mp,mech_ was 5.2 m s^−1^). Again, the U-shaped curve fitted the data better than the linear model (likelihood ratio test; *p* < 0.05). The model fitted to the data was

2.4 $$P_{\mathrm{met}}=3.73\;\times 10^{-4}\;U_{\infty }^{3}+\frac{2.52}{U_{\infty }}+0.490.$$

When we normalized power with the weight of bats the minimum weight-specific metabolic power was 7.1 W N^−1^ and maximum recorded weight-specific power (body mass (*M*_g_) × gravitational constant (*g*)) was 18.7 W N^−1^ (figure 2*b*). The model fitted to the data was

2.5 $$P_{\mathrm{met}/W}=4.11\;\times 10^{-3}\;U_{\infty }^{3}+\frac{28.6}{U_{\infty }}+5.90.$$

In both cases, the curves fitted to metabolic power were shallower than the curves fitted to mechanical power, indicated by the larger model coefficients corresponding to induced and parasite power. Minimum power speed estimated from metabolic flight power (*U*_mp,met_) was 6.9 m s^−1^, while maximum range speed (*U*_mr,met_) was 9.0 m s^−1^. The estimated *U*_mp,met/W_ was also 6.9 m s^−1^ while *U*_mr,met/W_ was 9.1 m s^−1^.

We estimated *P*_met_ from our measures of *P*_mech_ using the formula of Pennycuick [10]:

2.6 $$P_{\mathrm{met}}=1.1\left(\frac{P_{\mathrm{mech}}}{\eta }\;+\mathrm{BMR}\right),$$

which assumes a conversion efficiency (*η*) of 23% and incorporates respiratory and circulatory overheads (factor 1.1) and basal metabolic rate (BMR). BMR for the 10 individuals in this study averaged 0.08 ± 0.004 W, comparable to the value of 0.084 W predicted from allometry. Even with these additive costs, *P*_met_ was underestimated by 48% on average (36–62%) across all flight speeds.

### (c) Conversion efficiency

**Figure 3.**
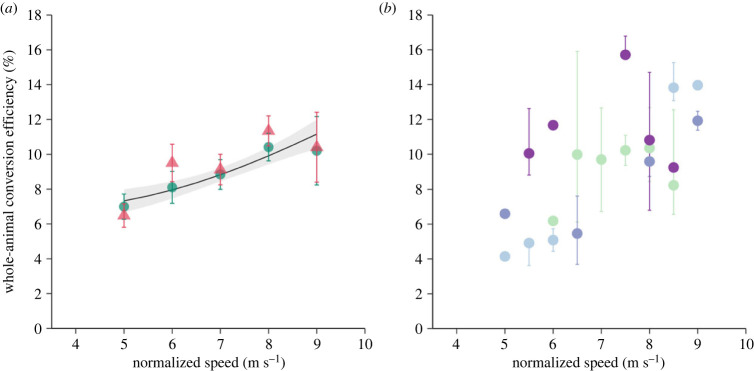
Conversion efficiency for *Pipistrellus nathusii* flying in a wind tunnel at air speeds rounded to the nearest 1 m s^−1^ between 5 and 9 m s^−1^. Efficiency data were calculated from (*a*) the average metabolic power and mechanical power across all bats at each air speed (triangles), the median metabolic power and average mechanical power across all bats at each air speed following the methods of von Busse *et al*. [25] (circles) or by dividing the estimates from the two models fitted to our empirical data (solid line and grey shading). Alternatively, conversion efficiency was estimated by (*b*) comparing all combinations of metabolic power and mechanical power per individual at air speeds within 0.5 m s^−1^ of each other, presented as the median efficiency (circles) and range of calculated efficiency (vertical bars) per bat (indicated by different colours). There was a significant positive correlation between conversion efficiency and air speed (*r*^2^ = 0.41, *p* < 0.01).

From our empirical data averaged across all individuals, we calculated whole-animal conversion efficiency to be approximately 9% for both raw and weight-/lift-corrected data (range 7.0–10.6%, electronic supplementary material, table S1), with an increase in conversion efficiency with increasing air speed (figure 3). Muscle conversion efficiency was only slightly higher, ranging from 8.4 to 12.5% and also increasing with air speed (electronic supplementary material, table S1). The increase in conversion efficiency with air speed was also evident when we calculated whole-animal efficiency using *P*_mech_ and *P*_met_ predicted from our models (figure 3*a*). When calculated following the methods of von Busse *et al*. [25] for comparison, which uses the median rather than the mean *P*_met_, conversion efficiency remained approximately 9%, with values ranging from 6.5 to 11.3%, again increasing with increasing air speed (figure 3*a*). This range overlaps previous results for the non-migratory bat *C. perspicillata* [25], and the data did not differ significantly when corrected for body mass and accounting for the effect of flight speed (*t*_6_ = −2.081, *p* = 0.08). When we focused on conversion efficiency at the individual level, comparing data for the four individuals where both *P*_mech_ and *P*_met_ were measured, we found a significant positive correlation between conversion efficiency and air speed (Pearson's correlation; *r*^2^ = 0.41, *p* < 0.001; figure 3*b*).

Our measurements for *P. nathusii* do not overlap the range of conversion efficiencies reported for the migratory bird species *S. atricapilla*, with a difference in group means of 10.9 percentage points (p.p.) (95% CI: 8.3, 13.5 p.p). This was true even when comparing mass-corrected values (see Methods; electronic supplementary material, figure S2), with a 4.5 p.p. and almost 45% greater mean mass-corrected conversion efficiency for *S. atricapilla* than *P. nathusii* (10.0 versus 5.5%, respectively). Unfortunately, the significant effect of speed on conversion efficiency in *P. nathusii* and the small sample size for *S. atricapilla* prohibited any meaningful direct statistical comparisons between these two species. However, there was no effect of speed on conversion efficiency in *C. atricapilla* (*t*_6_ = −1.65, *p* = 0.15) and a Wilcoxon rank-sum test revealed a significantly higher mass-corrected conversion efficiency in *S. atricapilla* than *C. perspicillata* (*W* = 0, *p* < 0.05).

**Figure 4.**
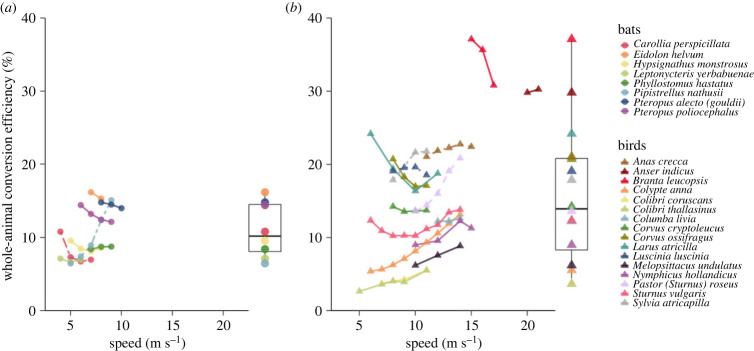
Estimated conversion efficiency for (*a*) 8 bat and (*b*) 16 bird species where data on metabolic rate in flight in wind tunnels at varying air speeds are available. Estimates of mechanical power were made using the *afpt* [12], and these were compared with data taken from the literature or this study (see electronic supplementary material, table S2 for study details). Scatter plots with corresponding lines indicate the mean conversion efficiency at each air speed for each species. Box and whisker plots were generated from the estimated conversion efficiency within ±1 m s^−1^ of minimum power speed as determined from metabolic power. Species are indicated by different colours, line types indicate the method of measuring metabolic power (solid lines—respirometry, dashed lines—sodium bicarbonate method, long dashes—mass loss, dotted lines—doubly labelled water).

Given that our results for *P*_mech_ most closely resemble those predicted by the *afpt*, we used this tool to more broadly investigate the variation in conversion efficiency between bats and birds for which data on metabolic rate in flight at various air speeds are available (electronic supplementary material, table S2). We calculated conversion efficiency by dividing measured *P*_met_ by the *afpt* estimated *P*_mech_ across flight speeds for 16 bird and 8 bat species (figure 4). Our estimates of conversion efficiency for *P. nathusii* only differed from our measured data by 1.6 p.p. on average (electronic supplementary material, table S3) and did not differ significantly from those calculated using the metabolic power curve for *P. nathusii* from Troxell *et al*. [5] (*t*_10_ = 0.54 *p* = 0.60). However, as air speed increased the error in estimate also increased, resulting in an overestimate of conversion efficiency of 4.9 p.p. at the maximum recorded flight speed. For *C. perspicillata*, conversion efficiency was only overestimated by 0.7 p.p. on average across four air speeds. Interestingly, peak conversion efficiency was estimated near minimum air speed for *C. perspicillata* and decreased with increasing speed. For the bird species *S. atricapilla,* where empirical data exist for only one speed at or above minimum power, our calculations again only underestimated conversion efficiency by 2.1 p.p. Across more than half of the species included in our meta-analysis, estimated conversion efficiency varied with air speed. In 10 species conversion efficiency increased with increasing air speed by more than 1 p.p. (1.3–8.6 p.p.). This trend was reversed in five species, with conversion efficiency decreasing with increasing air speed by between 2.3 and 6.3 p.p. The remaining nine species exhibited less than 1 p.p. change in conversion efficiency with air speed.

**Figure 5.**
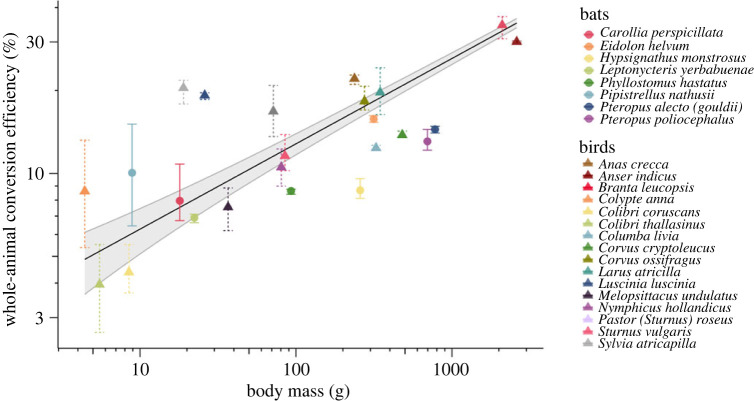
Variation in estimated whole-animal conversion efficiency with body mass in both bats (circles) and birds (triangles) presented on a log_10_ scale. Data are presented as mean conversion efficiency (circles/triangles) and range of data (vertical lines). There was a significant effect of log_10_-transformed body mass on estimated conversion efficiency (*z*_92_ = 3.78, *p* < 0.001), and this relationship did not differ between birds and bats (*z*_91_ = 1.13, *p* = 0.26). Solid line and shaded area represent results of the best-fit model ± 95% confidence intervals.

The absolute peak conversion efficiency was substantially higher for birds (37.1%) than bats (16.2%), which was likely a result of the significant effect of body mass on estimated conversion efficiency (*z*_92_ = 3.78, *p* < 0.001), with overall greater body mass and therefore estimated conversion efficiency occurring in birds (figure 5). The relationship between conversion efficiency and body mass did not differ between the two taxa when accounting for the effect of flight speed and species (generalized linear mixed model, glmm; *z*_91_ = 1.13, *p* = 0.26), with the group mean for birds only 4 p.p higher than for bats (95% CI: 1.85, 6.23). The predicted conversion efficiency for a bat of mean body mass flying at mean air speed from the fitted model was 9.7 ± 1.2% compared with 11.9 ± 1.1% for a bird with the same parameters. It is important to note that there was a large amount of uncertainty around our model coefficient for this variable (bird/bat; estimate (*β*) ± s.e. = 0.09 ± 0.08). The model fitted to the pooled data for conversion efficiency (*η*) was

2.7 $$\log_{10}\eta =0.24\log_{10}M+0.49.$$

The coefficient for body mass (*M*) varied with speed for each species with s.d. of 0.03, and the intercept varied between species with s.d. of 0.29.

When we focused on conversion efficiency near minimum power speed, we found an average of 11 ± 1.3% for bats versus 15.6 ± 2.4% for birds, and there was no significant difference between the two groups when accounting for body mass (*t*_20_ = 1.064, *p* = 0.3; figure 4). The overall variation in estimated conversion efficiency was greater in birds (range 3–37%) and mean peak conversion efficiency was 5% higher for birds than for bats (17.7 ± 2.2 versus 12.4 ± 1.4%), but again this difference was not statistically significant when accounting for body mass (*t*_20_ = 1.254, *p* = 0.22).

## 3. Discussion

By measuring both metabolic power and mechanical power in a single experiment on the same individuals, we show that whole-animal energy conversion efficiency is approximately 9% for Nathusius' pipistrelle, a small migratory bat from Europe. This result is consistent with the findings of von Busse *et al*. [25], the only prior study where conversion efficiency was measured empirically in bats, and suggests a lower conversion efficiency of flight in small bats compared with flight in small birds measured in the same manner as here [26]. Furthermore, our calculations of muscle conversion efficiency were only slightly higher (averaging approximately 11%) compared with the 30% reported for the similar bird study [26]. In addition, our findings challenge the traditional assumption that conversion efficiency can be treated as a constant for flying vertebrates, as we found variable efficiency across flight speeds. The results of our meta-analysis suggest that there is a strong scaling relationship between body mass and conversion efficiency, confirming recent findings [18,33,34]. Taken together, we are confident that our results accurately represent a lower conversion efficiency in some bats than the widely assumed 23% for vertebrate flapping flight [16,17].

While an overestimate of metabolic power could explain our calculations of low conversion efficiency, we are confident that our results are accurate for a number of reasons. Our measurements of metabolic power are in the same range as previous reports for *P. nathusii* in other wind tunnel experiments [5], and those predicted from allometry [35]. In addition, if we were incorrect in our assumption that bats oxidize solely glycogen, rather than a mix of glycogen and lipids, our metabolic power would be an underestimate rather than an overestimate. While our curve for *P*_met_ was shallower than previously reported for this species [5], potentially making our estimates of *U*_mp_ and *U*_mr_ less reliable, when we estimated conversion efficiency using the *P*_met_ curve of Troxell *et al*. [5], the results did not differ from our direct measurements (*t*_7_ = 0.91, *p* = 0.4) or measurements estimated from the *afpt* (*t*_10_ = 0.54, *p* = 0.60). Further, our estimates for mechanical power were greater than predicted by the flight models, refuting the possibility of an underestimate of mechanical power driving our low calculations of conversion efficiency.

Although the variation of conversion efficiency with flight speed has been suggested in the past (e.g. [36]) and was reconsidered in recent model-based estimates for bats and birds [18,22], it has yet to be validated by empirical data. Here, we demonstrate that conversion efficiency increased with increasing flight speed in *P. nathusii*, supporting the notion from previous studies that bats optimize muscle efficiency for flight at or above the maximum range speed [20]. This variation in conversion efficiency results from a steeper curve for mechanical power than metabolic power with increasing flight speed, which has also been shown in model-based estimates from birds [36]. We would predict conversion efficiency to plateau or even decrease when individuals fly at speeds much higher than maximum range speed, which are no longer optimal for muscle efficiency. From our meta-analysis estimates, it appears that *P. nathusii* is unique among bats studied in terms of the increase in conversion efficiency with speed (electronic supplementary material, figure S3), and this phenomenon was more common among the birds considered in our study. Peak conversion efficiency may not be tuned toward maximum range speed in all species, perhaps indicating some ecological distinctions. In the fruit-eating bat *C. perspicillata*, our estimates suggest peak conversion efficiency may be at lower speeds, which may reflect selection for optimal performance associated with foraging ecology. This may also explain the relatively low conversion efficiency values reported for this species in the past. Given these findings and the known limitations of modelling mechanical power at low flight speeds, we advocate that future studies consider direct measurements above maximum range speed and below minimum power speed, to further investigate this pattern.

When compared with previous empirical findings, our results suggest that the capacity for maximizing conversion efficiency is higher in a small bird than the small bats studied even when body mass is considered. This could be due to fundamental phylogenetic differences between bird and bat physiology or may reflect the varied ecology and life histories of the few species that have been studied thus far. This is indicated, at least to some extent, when we compare the available measured data with predicted values from our meta-analysis model (see electronic supplementary material, table S3). For the two migratory species, measured conversion efficiency was higher than predicted for their size across all flight speeds; by 2.7 ± 0.5 p.p. on average for *P. nathusii* and 12.8 ± 0.5 p.p. on average for *S. atricapilla*. On the contrary, predicted conversion efficiency generally overlapped with measured data for the non-migratory *C. perspicillata*, differing by 0.3 ± 0.8 p.p. on average (electronic supplementary material, table S3). More empirical data are needed to better ascertain whether or not these differences reflect adaptations associated with migratory behaviour, or are related to limitations of available data that potentially exclude peak conversion efficiency for some species.

When we considered these results in the context of our broader meta-analysis, however, we found no discernible differences between birds and bats. This was also true for the scaling of body mass and conversion efficiency. Our analysis was limited to only 24 species and the s.d. around our coefficient for taxa was large, and thus we cannot rule out the possibility of different scaling relationships between birds and bats. This is pertinent considering that the factors constraining body mass in birds and bats differ, with probable implications for conversion efficiency. A positive scaling relationship between body mass and muscle efficiency has been described for birds from both intraspecific [33] and interspecific studies [37], and it has been suggested that some limiting plateau may be reached in large species [34], possibly related to circulatory constraints [37]. Potentially for this reason, Guigueno *et al*. [18] fitted two separate phylogenetic generalized linear models to determine the relationship between body mass and conversion efficiency for 22 bird and 13 bat species. Interestingly, they found that the inclusion of BMR as an additive factor to flight costs resulted in more parsimonious models, supporting previous models that include a factor for BMR when estimating *P*_met_ from *P*_mech_ [10].

In bats, different aspects of physiology, flight aerodynamics, echolocation and their interactions constrain body mass evolution along the size scale [38,39]. Importantly, the flight muscle mass in bats is proportionately smaller than those of birds, with downstroke muscles making up only approximately 9% of the body in comparison with approximately 15% on average for birds [40]. This, coupled with wingbeat frequency constraints, puts the maximum estimated attainable body mass for bats at near 2 kg, compared with around 20 kg for the largest extant bird species capable of flapping flight [40]. Bats not only have smaller downstroke muscles but also possess a greater array of small muscle fibres that extend along the arm and forearm, as well as within the wing membrane [41–44]. We speculate that supplying enough blood to a few large, centralized muscles close to the heart and lungs could potentially be more effective than supplying blood to many small muscles that are more widely dispersed, and potentially more energetically demanding. If this is the case, it may result in reduced efficiency of energy supply in bats when compared with birds. This could potentially help to explain previous findings of an additive effect of BMR to flight costs in bats but not birds [18]. In addition, the wing membranes of bats are bare, to dissipate high heat loads generated in flight [45], which results in convective cooling of the muscles embedded in the wing by up to 12°C during flight [46]. While these muscle fibres are specialized to function at lower temperatures [47], the blood supplied to them is cooled, which increases blood viscosity and potentially reduces efficient oxygen transport [48], also impacting the overall costs of flight.

The highly articulated nature of the bat wing, and thus greater diversity of flight muscles, aids in fine-scale adjustments of wing shape, flapping motion and membrane stiffness during flight—factors affecting lift generation and manoeuvrability [42–44]. Increased manoeuvrability is important for aerial insectivores as they must capture their small insect prey in flight, and insectivory in general has been suggested to constrain body mass in both birds and bats [38,40]. Manoeuvring is therefore an important contributor to overall flight costs [49], which may influence the variation in conversion efficiency between species. We suggest that the selection pressure for effective manoeuvrability to aid prey capture in insectivores potentially outweighs the pressure for superior flight efficiency, possibly even in migratory species. During long-distance flights migratory birds primarily oxidize fatty acids, and as mammals bats are less capable of mobilizing and oxidizing this fuel [50], relying primarily on exogenous sources and stored glycogen [51]. This means migratory insectivorous bats must forage along their journey [52], making manoeuvrability for foraging a powerful selection pressure on flight performance. This may help to explain why our findings for *P. nathusii*, the bat with the world's longest documented migratory distance, were still low in comparison with the migratory bird *S. atricapilla* even when we corrected for body mass. *Pipistrellus nathusii* is the only insectivorous bat for which reliable data on metabolic rate in flight at multiple speeds exist, and we advocate that more species be considered in order to further investigate the ecological components influencing conversion efficiency.

Our findings have important implications for the modelling of flight costs in small migratory species. As many migratory species are elusive, we often rely on simulations and models to predict where and how they travel. Current model assumptions result in underestimates of metabolic power by up to 62% for *P. nathusii*, and similar underestimates are expected for other small species. Extrapolating these findings to overall costs of migration could result in gross overestimates of migration distance. If conversion efficiency in other species is indeed much lower than the presumed 23%, the ability for small migrants to cover large distances must require their ability to offset high flight costs via additional means. It is likely that both refuelling, via foraging in flight, and/or using torpor during daytime stopovers [53], is much more important to migration energy budgets than currently accounted for. Our findings therefore also suggest that torpor-assisted migration may be essential for small migratory species of less than 10 g, such as bats and hummingbirds. Consequently, the preservation of intact foraging habitat along their migratory route is crucial, alongside adequate roosting structures that enable maximal energy savings from torpor.

## 4. Conclusion

Our direct measurements suggest that conversion efficiency in some bats is lower than the presumed 23% for bird flight. Our meta-analysis revealed that this is primarily related to the body mass of the species studied and supports previous findings suggesting efficiencies deviating from 23% [22,34]. However, our results also indicate that ecological pressures may induce additional variation between species and across flight speeds, as has been previously suggested [34]. More empirical data are needed to determine if this is the case and could provide important insight into maximum conversion efficiency as it relates to more ecologically relevant flight speeds. We found that the *afpt* [12] provided better estimates of mechanical power than Pennycuick's *Flight* [10], supporting a previous study of two other bat species [15]. While we advocate that the best-practice scenario involves measurement of both metabolic power and mechanical power empirically, we acknowledge the practical difficulties of this methodology and therefore suggest that when estimates of mechanical power are made, the *afpt* most closely resembles true mechanical flight power. As a direct consequence of our findings, we would therefore also recommend that when using flight models to calculate flight costs the conversion efficiency should be adjusted for body mass of the focal species accordingly.

## 5. Methods

### (a) Study species and training

We captured 10 adult *P. nathusii* (4 male, 6 female; body mass at capture 7.6 ± 0.8 g) in September 2018 from Pape, Latvia under the licence (no. 31/2018) from the Latvian Nature Conservation Authority. Individuals were then transported to Lund University, Sweden for the experiments (see electronic supplementary material). Experiments were conducted in the wind tunnel facility of Lund University. After a one-week period of acclimation to captivity, we began training individuals to fly in the wind tunnel. All experiments were performed in accordance with ethical guidelines for animal experimentation and approved by the Malmö–Lund animal ethics committee (5.8.18-10124/2018).

### (b) Metabolic power measurements

For measuring the metabolic rates in flying bats, we used the ^13^C-labelled sodium bicarbonate method as outlined in Hambly & Voigt [31]. Animals were placed in a 500 ml respirometry chamber through which CO_2_-free air passed at a constant rate of 470 ml min^−1^ STP. After measuring the baseline isotopic enrichment of exhaled breath over a period of at least 5 min, a bat was taken out of the chamber and injected intra-peritoneally with a 100 mg isotonic dosage of ^13^C-labelled sodium bicarbonate (0.29 mol l^−1^, Eurisotop, Saarabrücken, Germany) and placed back into the respirometry chamber.

We measured the enrichment of ^13^C in the exhaled breath of animals using a flow-through cavity ringdown laser spectrometer (LGR-ICOS Carbon Dioxide Isotope Analyzer, ABB–Los Gatos Research, San Jose, USA). After complete equilibration, tracer enrichment declined exponentially, at which time we transferred the bat from the respirometry chamber into the wind tunnel, where it flew for approximately 1 min (66 ± 9 s). Timing of flights (start, stop and all landings) was recorded on a stopwatch and via video recordings, which were synchronized. After the end of a flight trial, we returned the bat to the chamber to further record the clearance of the marker for at least 15 min. After the experiment, we transferred the bat back into the maintenance cage. For converting *V*_CO2_ into metabolic power in watts (W), we assumed that bats oxidized predominantly glycogen during the 1 min flight interval and thus used a conservation factor of 21.1 J ml^−1^ CO_2_ [54]. If we assumed that bats oxidized a mixture of 80% carbohydrates and 20% lipids (respiratory quotient, RQ = 0.88) using the conversion factor 23.7 J ml^−1^ CO_2_, our estimates of metabolic power would increase by 11%. We also determined the basal metabolic rate (BMR) of these bats using flow-through respirometry at ambient temperatures between 28 and 35°C. For further details, see the electronic supplementary material.

### (c) Mechanical power measurements

Mechanical power was estimated for four individuals, with an average of 12.5 sequences per individual and an average of 3.6 wingbeats per sequence. Power was calculated from the rate of kinetic energy added to the wake by the bats as suggested by von Busse *et al*. [25]. The power was calculated from the velocity fields measured using tomographical particle image velocimetry (TomoPIV) in a thin volume perpendicular to the free stream flow of the wind tunnel, following Johansson *et al*. [6]. In addition to the method described in previous studies, we performed a background flow subtraction and a masking procedure to improve the power estimate. Systematic variation in the background flow was estimated using all sequences and used to make the background flow homogeneous. We also used a volumetric masking of the wake, based on thresholding three-dimensional vorticity magnitude (as described in [55]), to isolate the wake structures originating from the bats from the background. Using the vorticity, we then reconstructed the flow field, according to von Busse *et al*. [20]. For details about the set-up and calculations, see the electronic supplementary material.

In addition to estimating power, we calculated the net drag (*D*_net_) in the wake using a wake deficit model [6]:

5.1 $$D_{\mathrm{net}}=\rho {\int \int }_{\mathrm{wake}\;\mathrm{area}\;}^{\;}u(y,z)\;(U_{\infty }(y,z)+u(y,z))\;dydz.$$

For steady flight, the net drag during a sequence is expected to be zero, but our data showed a systematically higher net drag. The net drag can be viewed as either a result of the animal not maintaining speed or an overestimation of the background speed. Given our multi-step procedure in estimating the background speed and the fact that the bats flew steadily for several wingbeats, it is likely to be the latter explanation. To compensate for this, we added the power corresponding to the net drag (*D*_net_*U*) to our estimated power.

To make sure that the flights were stable we also calculated the vertical force by integrating the in-plane vorticity (*ω_x_*) multiplied by the horizontal distance to the centre of the body wake (*y* − *y*_0_) over the measured wake after applying the three-dimensional mask to the data and compared with the weight of the animals:

5.2 $$F_{v}=\rho U_{\infty }^{\;}(y,z){\int \int }_{\mathrm{wake}\;\mathrm{area}\;}^{\;}(y-y_{0})\omega_{x}(y,z)\;dydz.$$

The metabolic and mechanical power measurements were not made simultaneously since the ^13^C-labelled sodium bicarbonate method is very time-sensitive, and flight behaviour of the bats can be influenced by the laser. To account for variation in weight and that the metabolic measurement flights were longer and presumably more stable than the mechanical power measurement flights, we calculated the weight- and lift-specific power, respectively. This method has previously been shown to reduce variation in the data [26], as expected owing to the power dependency of lift in both *P*_ind_ and *P*_pro_ [12].

### (d) Mechanical power modelling

In addition to our measurements, we estimated the mechanical power using two models for animal flight, Pennycuick's *Flight* [10] and the *animal flight performance tool* (R-package *afpt*; [12]). In the models we used the following settings: mass (*M*) = 0.089 kg, wing span (*b*) = 0.23 m, wing area = 0.0077 m^2^, wingbeat frequency (*f*_wb_) = 9.9 Hz and body drag coefficient (*C*_db_) = 0.4. Each bat was photographed with the wing outstretched together with a reference length, from which wing span and area were measured using ImageJ (v.1.50i). The body drag coefficient was selected to follow previous studies [15,22,56].

### (e) Data analysis/selection criteria

For the metabolic power measurements, only data where washout curves and *V*_CO__2_ were stable during pre- and post-flight periods were analysed. From these data, we only selected flights where the total amount of time bats landed during the flight period was less than 40% of the flight time, meaning that the actual time spent in flight ranged from 60 to 100% of the total flight period (mean = 89 ± 2%). The ^13^C-labelled sodium bicarbonate method accounts for the time that animals were not flying by assuming that all flight occurred in the middle of the time period within the wind tunnel, extrapolating from resting data either side. Previous calculations of flight power using this method were considered accurate when animals flew as little as 51% of the time [57]. Outliers were classified as any value with a *Z*-score exceeding 2 (calculated by subtracting the mean and dividing by s.d. of flight costs at a given speed). Finally, only individuals where we had sufficient data across a range of flight speed (more than three flights) were included in the final analysis. This reduced the number of viable flights from 81 to 42.

For the mechanical power measurements, we only included sequences that contained more than half of the wake for more than one wingbeat and that had a wingbeat-averaged vertical force deviating no more than ± 20% from the weight of the animal (measured prior to the flight). For sequences that contained half the wake, vertical force estimates were doubled and the wake was mirrored in the centre plane of the body prior to the Helmholtz–Hodge decomposition and power estimate.

To control for differences in weight between individuals, which is expected to affect the characteristic flight speed according to *M*_g_^1/6^ [10], we used normalized flight speeds for the statistical analyses. We did this following the methods of [58], where normalized flight speed for a certain individual was defined as

5.3 $$U^{*}\;=\;U{\left(\frac{M_{\mathrm{g-ref}}}{M_{g}}\right)}^{1/6},$$

where *M*_g_ is the average weight of the individual and *M*_g*-*__ref_ is the average weight of all bats across the study.

We fitted a mixed effect model using the package *nlme* [59] with individual included as a random factor to the data. To estimate minimum power speed, we used the *afpt* given the parameters for body morphometrics mentioned previously.

### (f) Conversion efficiency calculations

Whole-animal conversion efficiency (*η*) was calculated by dividing mechanical flight power (*P*_mech_) by metabolic flight power (*P*_met_) using the following equation:

5.4 $$\eta =\frac{P_{\mathrm{mech}}}{P_{\mathrm{met}}}.$$

We used four different approaches to perform this analysis: (i) taking the average *P*_mech_ and *P*_met_ across all individuals from data where air speed was rounded to nearest 1 m s^−1^; (ii) using the methods of von Busse *et al*. [25], where average *P*_mech_, rounded to the nearest integer air speed (m s^−1^), was divided by the median *P*_met_ at that same speed; (iii) dividing estimates of *P*_met_ by estimates of *P*_mech_ taken from our fitted models; and (iv) comparing the data from the four bats where both *P*_mech_ and *P*_met_ were measured at similar air speeds. For this final calculation, we compared data where the measured air speeds were within ±0.5 ms^−1^ of each other. This resulted in the duplication of either *P*_mech_ data (*n* = 31) or *P*_met_ data (*n* = 59) used in calculations, introducing increased variation and autocorrelation in our results. However, because the flight activity of the bats varied throughout the measurements, we cannot be sure that all bats flew at the exact air speed that was measured and have no reliable estimate of measurement error in air speed. We believe that this approach enables us to address some of this uncertainty, and we therefore present our calculations of conversion efficiency as median ± range per bat per average speed rounded to the nearest 0.5 m s^−1^.

To assess the relationship between air speed and conversion efficiency, we performed a Pearson's correlation on our calculations from the four individuals where both *P*_mech_ and *P*_met_ were measured within ±0.5 m s^−1^ of one another, where air speed was averaged between the two measurements. We chose this approach because data independence is not an assumption of the model, and owing to duplications in our method, our data cannot be considered independent.

Muscle conversion efficiency (*η*_fm_) was calculated using the following equation from Morris *et al*. [21]:

5.5 $$\eta_{\mathrm{fm}}=\frac{1.1}{\left(P_{\mathrm{met}}-\mathrm{BMR}\right)}P_{\mathrm{mech}},$$

where BMR is basal metabolic rate measured for these bats (see electronic supplementary material for details).

### (g) Comparing our empirical data with literature data

Data were collected from the literature for bats and birds of different body sizes where data were available for flight metabolic rate at a range of air speeds in a wind tunnel. This resulted in a dataset of 16 bird and 8 bat species with body masses between 0.004 and 2.6 kg (electronic supplementary material, table S2). Metabolic power from these studies was primarily measured using respirometry; however, additional methods, including the ^13^C-labelled sodium bicarbonate method, doubly labelled water and mass loss, were also included. A recent meta-analysis [18] found no difference between metabolic power measured via these different methods and therefore we considered all data to be comparable. Data were derived either from tables within each paper, or from equations of metabolic power versus wind speed, or were digitized from summary figures using the software PlotDigitizer [60]. Data for oxygen consumption were converted to watts (W), assuming the oxyjoule equivalent of 20.1. Estimations of mechanical power in flight were calculated using *afpt* [12] with body mass, wingbeat frequency and morphometrics from the metabolic rate study (where available). When morphometric data were not available, they were collated from additional sources (primarily [61,62]); see electronic supplementary material, table S2 for all input data. We only included metabolic power data above the minimum power speed calculated from *P*_met_, as below this point estimates of *P*_mech_ are less accurate. As a second method of quality assurance we also excluded any estimated *P*_mech_ data marked by the *afpt* with a ‘red flag’. The *afpt* provides four ‘red flag’ indicators for speed, thrust and frequency, to highlight data that are outside the model range and cannot be considered accurate.

To assess the relationship between conversion efficiency and body mass, we log_10_-transformed both variables and applied generalized linear mixed effects models using the R-package *glmmTMB* [63]. We accounted for the effect of speed on our data by including speed as both a fixed effect and a random effect, so as to allow for fitting of random slopes and to account for the different relationship between speed and conversion efficiency per species. We also included the classification of each species as either ‘bird’ or ‘bat’ in the model as a fixed effect, and species as a random factor. We also compared peak conversion efficiency or conversion efficiency at minimum power speed between birds and bats by performing general linear models in the R-package *stats* (https://www.R-project.org/). For this analysis, we included the interaction between body mass and species classification (bird/bat) to account for the effect of body mass on conversion efficiency.

To compare our empirical data with other empirical data from the literature, we used general linear models or, in the case of small sample sizes, Wilcoxon rank-sum tests using the *stats* package in R. In order to account for the effect of body mass when comparing different species, we transformed conversion efficiency to ‘mass-corrected’ values by dividing each value by *M*^0.24^ and included the classification of each species as either ‘bird’ or ‘bat’ as a fixed factor in our analyses. The exponent for the mass-corrected values was derived from our model of conversion efficiency against body mass. To compare our data with previous reports for *P. nathusii*, we used general linear models of conversion efficiency against speed, including ‘data source’ as a fixed factor.

## Data Availability

The data are available from the Dryad Digital Repository: https://doi.org/10.5061/dryad.6q573n631 [64]. Supplementary material is available online [65].
